# Contributions of Speed and Accuracy to Translational Selection in Bacteria

**DOI:** 10.1371/journal.pone.0051652

**Published:** 2012-12-14

**Authors:** Wenqi Ran, Paul G. Higgs

**Affiliations:** 1 Department of Physics and Astronomy, McMaster University, Hamilton, Ontario, Canada; 2 National Center for Biotechnology Information, National Library of Medicine, National Institutes of Health, Bethesda, Maryland, United States of America; Cairo University, Egypt

## Abstract

Among bacteria, we have previously shown that species that are capable of rapid growth have stronger selection on codon usage than slow growing species, and possess higher numbers of rRNA and tRNA genes. This suggests that fast-growers are adapted for fast protein synthesis. There is also considerable evidence that codon usage is influenced by accuracy of translation, and some authors have argued that accuracy is more important than speed. Here we compare the strength of the two effects by studying the codon usages in high and low expression genes and on conserved and variable sites within high expression genes. We introduce a simple statistical method that can be used to assess the significance and the strength of the two types of bias in the same sets of sequences. We compare our statistical measure of codon bias to the common used codon adaptation index, and show that the new measure is preferable for three reasons for the purposes of this analysis. Across a large sample of bacterial genomes, both effects from speed and accuracy are clearly visible, although the speed effect appears to be much stronger than the accuracy effect and is found to be significant in a larger proportion of genomes. It is also difficult to explain the correlation of codon bias in the high expression genes with growth rates and numbers of copies of tRNA and rRNA genes on the basis of selection for accuracy. Hence we conclude that selection for translational speed is a dominant effect in driving codon usage bias in fast-growing bacteria, with selection for accuracy playing a small supplementary role.

## Introduction

Translation is the process by which ribosomes synthesize proteins in cells. Protein synthesis is essential to all organisms, and cells expend a large amount of energy and time on translation. For single celled organisms, such as bacteria, there is a direct relationship between the rates of cellular processes such as translation and the rate of cell growth and cell division. Therefore, improvements in translation should increase the fitness of the organism. The term ‘translational selection’ refers to selection to optimize the translation process itself rather than selection acting on the functions of the proteins produced by translation. One of the main pieces of evidence for translational selection is the observation that the choice of synonymous codons appears to be influenced by selection in many organisms. Synonymous changes in the gene do not affect the resulting protein but can affect the way that the mRNA is translated by the ribosome.

The speed of translation is one of the key factors on which translational selection can act. Speed has the direct benefit that the proteins required are produced faster, and the secondary benefit that if a given ribosome finishes translation of one sequence, it can begin work on another. Hence, speeding up translation means that the same total protein production rate can be achieved with fewer ribosomes. Synthesis of the ribosomal proteins and RNAs themselves is costly to the cell, so getting the most out of a limited number of ribosomes is important for efficiency. The argument for translational speed/efficiency explains the observation that codon usage is most strongly biased in a relatively small number of genes that are highly expressed in conditions of rapid growth [1]. In *E. coli*, the concentrations of tRNAs are also found to vary with growth conditions [2] and are found to correlate with the frequencies of codons in highly expressed genes.

Ribosomal proteins and translational elongation factors are among the most highly expressed genes in bacteria, and are easily recognizable conserved genes in most genomes. These genes are often used as a reference set, and the codon frequencies in these reference genes are used to define measures of codon bias with which to compare the strength of translational selection in different genes. The first of these is the codon adaptation index (CAI), introduced by Sharp and Li [3]. However, codon frequencies can also vary due to mutational biases as well as because of selection. More recent work has used population genetics theory to predict the way that codon frequencies should vary under both mutation and selection, and hence to develop measures of codon bias that distinguish the strength of selection from the underlying mutational bias [4], [5], [6], [7]. These methods look at the difference in codon frequencies between high and low expression genes, rather than simply at the frequencies in the high expression genes. Another measure of translational selection is the tRNA adaptation index (tAI) that weights codons according to how well they match the pool of tRNA genes [8]. However, to do this accurately requires knowledge of the relative rates of pairing of different anticodon-codon combinations, and our own studies [7] have shown that this is a complex issue that goes beyond the simple wobble rules.

Further evidence for the importance of translational speed in bacteria is the observation that codon bias is strongest in organisms that have fast growth rates [9]. These same fast-growing organisms are also found to have larger numbers of duplicated copies of tRNA genes [9] and larger numbers of copies of ribosomal RNA operons [5]. Our interpretation is that rapid growth requires rapid translation and hence a high rate of production of rRNAs and tRNAs. This is facilitated by duplication of the RNA genes. There is direct experimental evidence that when mixtures of bacteria are grown in culture together, the colonies that appear most rapidly are those which have the largest number of rRNA operons [10]; thus, having duplicated rRNAs allows a rapid growth response in conditions where food is plentiful. We have shown that selection for translational efficiency can favour genomes with increased numbers of tRNAs and can lead to coevolution of tRNA content and codon usage [6], [7].

Bacterial genomes usually do not have large non-coding regions and, in general, duplicated genes are rare. This suggests that the efficiency of DNA replication is also important to bacteria and this keeps genomes from becoming larger than necessary. The fact that tRNA and rRNA genes *are* often duplicated attests to the importance of these genes. It is interesting to note that ribosomal proteins, which are required in cells in equally high numbers as ribosomal RNAs, usually have single-copy genes. High levels of proteins can be achieved by optimizing translation from a limited number of mRNAs, whereas high levels of rRNAs and tRNAs can only be achieved by duplicating the genes, and hence increasing transcription.

The other important aspect of translational selection is accuracy. Occasional mis-pairings between codon and anticodon may occur during translation, leading to errors in the protein sequence. This is wasteful, if the protein is no longer functional, and may actually be harmful, if mistranslated proteins misfold to structures that are toxic, as has been suggested [11]. If errors in translation are sufficiently frequent and sufficiently harmful, and if the error rate differs among synonymous codons, then selection may chose codons that have the lowest error rate. A signature of selection for accuracy is that codon frequencies differ between conserved and variable sites within the same genes [12], [13]. It is presumed that sites that are evolutionarily conserved between species are particularly important for protein function. Thus, accurate translation of these sites should be particularly important, and the frequency of the most accurate codons should be higher at the conserved sites.

There seems to be clear evidence that both the speed and accuracy of translation can differ between synonymous codons. We have previously discussed many of the specific details of codon-anticodon interactions that influence which codons are preferred as a function of which tRNAs are present in an organism [7]. We also reviewed the way in which modified bases on the tRNA influence translational speed and the ability of tRNAs to distinguish between correct and incorrect codons. Our theoretical interpretation of the codon frequency data [6], [7] has been primarily in terms of selection for speed; however, given the evidence that accuracy is also important, it is of interest to look for evidence of codon bias due to selection for both accuracy and speed in the same gene sequences and the same organisms. In this paper we will develop a statistical test to detect differences in codon frequencies between any two sets of codons, and to measure the extent of these differences. We will apply this test to the comparison of codon frequencies in high and low expression genes, and to the comparison of codon frequencies in conserved and variable sites within high expression genes. By comparing these factors in the same set of organisms, we are able to make a useful comparison of the two main causes of translational selection across many species.

## Methods

### Sequence Analysis

For ease of comparison with previous papers, we use the same set of 80 widely distributed bacterial species used by Sharp *et al.* [5] and our previous studies [6], [7]. Gene sequences from these species were aligned and codons were recounted independently of the previous papers. Initially, 54 ribosomal protein genes and 3 elongation factors of *Escherichia coli* were downloaded from the *E. coli* database. BLAST was used to find the orthologous protein sequences in each of the other genomes, where possible. We wished to include only sequences that are conserved across a large majority of genomes and that can be reasonably assumed to be high expression genes in all cases. Hence, sequences were excluded if the *E* value of BLAST for the best match was larger than 0.05, or if the best matching sequence was more than two times longer than the *E. coli* sequence. If a reliably matching sequence was found in at least 73 species, the gene was retained as part of the high expression data set. Otherwise the gene was excluded for all species. This resulted in retention of the following 47 high expression genes whose sequences could be located in almost all species: L1–L7/L12, L9–L11, L13–L22, L24, L27, L28, L31, L35, S2–S20, EF–G, EF–Tu, EF–Ts. Finally, the two species *Clostridium tetani E88* and *Mycoplasma penetrans HF-2* were excluded because a reliably matching sequence could not be determined for more than 1/3 of the original 57 genes. Having determined the set of genes and species, the protein sequences were aligned for each gene using MUSCLE [14]. The codon-based alignments of the DNA sequences were constructed to be consistent with the protein alignments.

For purposes of comparison of high and low expression genes, the codon counts summed over the 47 aligned genes were counted as the high-expression data set, and the codon counts summed over all other genes in the genome were treated as the low-expression set. Although a small number of other genes may have expression levels comparable to the ribosomal proteins and elongation factors, these contribute very little to the total codon count in the rest of the genome, and this codon count is dominated by the large majority of genes whose expression level is much less than that of the ribosomal proteins.

For the purposes of comparison of conserved and variable sites, we identified the conserved sites within the alignments of the high expression genes in the following way. The most frequent amino acid at each site was determined from the alignment. There are very few sites for which the amino acid is 100% conserved in every species; therefore a strict definition of conserved sites is not possible. We counted a site as conserved if the fraction of species possessing the most frequent amino acid was at least equal to a specified value *f_min_*. Otherwise the site was counted as variable. We varied *f_min_* in the range 60–90%, and we used 80% for most of the results in this paper. The conserved codon counts are obtained from summing codons for the most frequent amino acid at the conserved sites. The variable codon counts are obtained by summing all codons at variable sites plus the codons for the less frequent amino acids at the conserved sites.

### Quantification of Codon Bias

The method given here is a general method for determining to what extent the codon frequencies in two data sets differ. We suppose that two sets of sequences (or two sets of sites within sequences) have been identified, which we will call A and B. For example, A and B could represent the high and low expression genes, or the conserved and variable sites, or any other two sets of codons. The number of occurrences of each codon *i* in sets A and B are denoted 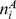 and 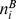. From this, the relative frequencies of codons for each amino acid in each set are
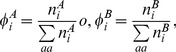
(1)Where 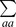 means a sum over codons for the same amino acid as codon *i*. The average codon frequencies in the combined sets are



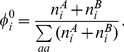
(2)We can now use a maximum likelihood (ML) method to develop a statistical test for difference in frequencies between sets A and B. Firstly we make a null hypothesis that the frequencies in both groups are same. The ML estimators of the frequencies are equal to the observed average frequencies 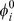. The log likelihood according to the null model is
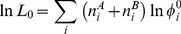
(3)


Here the sum is over all codons except for stop codons and the single codons for Met and Trp. We then consider an alternative model in which codon frequencies are allowed to be different in the two groups. The ML estimators of the frequencies in the two groups are then given by 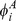 and 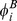, and the log likelihood is
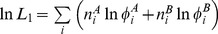
(4)


A standard likelihood ratio test can be used to determine whether the alternative model is a significant improvement on the null model. If the null model is true, the quantity 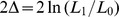 should have a χ^2^ distribution with a number of degrees of freedom equal to the difference in the numbers of degrees of freedom of models 0 and 1. For each amino acid the number of degrees of freedom is one less than the number of synonymous codons. In the standard genetic code there are nine amino acids with two codons, one amino acid with three codons, five amino acids with four codons and three amino acids with six codons. Hence the number of degrees of freedom in the likelihood ratio test is 9×1+1×2+5×3+3×5 = 41. By calculating the *p* values from theχ^2^ distribution, the values of 2Δ can be used to determine whether codon frequencies are significantly different between sets A and B.

The significance of this test depends on the total number of codons in each data set. Small differences in frequencies will show up as significant if the data sets are large. In order to compare the strength of codon bias in different cases with different data sets (e.g. different species) it is useful to define
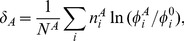
(5)where *N^A^* is the total number of codons in set A. The quantity *δ_A_* is the improvement in the log likelihood per codon in set A obtained when the A set is treated separately rather than as part of the average. This is a measure of the strength of codon bias in the A set relative to the average. If we consider the specific case where set A is the high expression genes (H), then *δ_H_* is a measure of the strength of selection for translational efficiency, whereas if set A is the conserved sites (C), then *δ_C_* is a measure of the strength of selection for translational accuracy.

Another usage of *δ* is to compare the strength of translational selection in different sequences in a same organism. If we consider one single sequence with codon counts 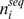 and total number of codons *N_seq_*, then the quantity
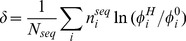
(6)measures how well optimized is the codon usage in that sequence. This will be positive for the ribosomal proteins and elongation factors in the high expression set from which the 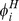 frequencies were calculated, and also for any other strongly biased genes. This is similar to what is usually done with the codon adaptation index, CAI [3]. For CAI, the weighting factor for each codon is 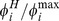, where 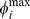 is the frequency of the most frequent codon in the same codon family in the high expression genes. The CAI is defined as a geometric average of these weighting factors, but the logarithm of the CAI is an arithmetic mean:



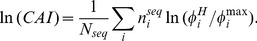
(7)Both *δ* and CAI will have high values for sequences whose codon usage matches that of the ribosomal proteins, thus they will be highly correlated. We compare values of *δ* and CAI later in the paper. Roth *et al.* [15] have reviewed a large number of other measures of codon usage bias, and they have also pointed out the similarity between the CAI and the likelihood ratio. In particular, they discuss a codon preference measure *P* that uses weighting factors of the form 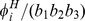, where the *b*’s are the individual base frequencies. This formula is similar, but it is not suitable for our purposes because even the low expression genes do not have codon frequencies that are equal to the product of the three base frequencies. The purpose of our measure *δ* is to detect genes that have codon bias due to selection for translational efficiency, and for this purpose, the weighting factors in Eqn. 6 are most appropriate.

## Results

### Statistical Significance of Codon Biases

In this section we use statistical tests to detect the presence of selection on codon usage in high expression versus low expression genes (HL comparison) and in conserved versus variable sites (CV comparison). The HL effect is very easy to detect in individual codon families. The simplest example is codon families with two codons ending in U and C (for example the UUU and UUC codons for Phe). The codon counts for the H and L set form a 2×2 table. The null hypothesis that the frequencies are equal in the two sets can be tested using a simple χ^2^ test with one degree of freedom. This test was carried out on all U+C codon families in all genomes. The value of χ^2^ was significant at the 5% level in 80.4% of codon families across the range of genomes. In comparison, the difference between the conserved and variable sites is less marked, and was significant at the 5% level in 12% to 15% of codon families, depending on *f_min_* (see Table 1). This demonstrates that an effect exists in the CV comparison, because only 5% of cases would be significant due to chance alone. This conclusion does not depend greatly on the choice of *f_min._* There appears to be a small difference between C and V sites that is difficult to detect in single codon families. This motivates the use of the likelihood ratio test described in the methods section. By combining the codon data from all codons simultaneously, this test is more powerful than the χ^2^ test on individual codon families.

**Table 1:** Percentage of two-codon U+C families that show significant codon frequency differences between high and low datasets and conserved and variable datasets.

Dataset	Percentage of cases with *p*<0.05
HL	80.4%
CV *f_min = _*90%	12.0%
CV *f_min = _*80%	14.5%
CV *f_min = _*70%	12.6%
CV *f_min = _*60%	13.6%

**Figure 1:**
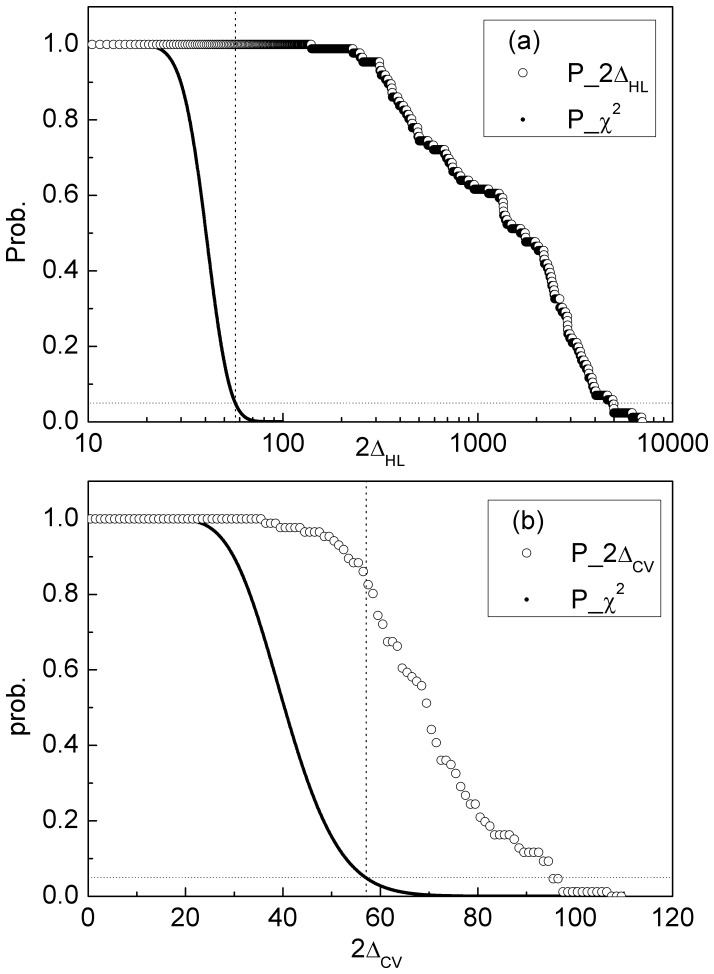
Cumulative probability distribution of 2Δ. Distributions are shown for our data (symbols) and for the χ^2^ distribution expected in the null hypothesis (solid line). (a) For the HL comparison, the distribution of 2Δ is very much different than expected under the null hypothesis. 100% of species have a 2Δ value that is significant at the 5% level (shown by the vertical dashed line). (b) For the CV comparison, the distribution also differs noticeably from the null hypothesis, with 80% of species having a 2Δ value significant at the 5% level.

The likelihood ratio statistic 2Δ was calculated as in the methods section for each species for both HL and CV comparisons (using *f_min_* = 80%). The cumulative probability distributions of 2Δ are plotted in Fig. 1 (i.e. the *y* axis shows the fraction of species that have a 2Δ value greater than or equal to the value on the *x* axis). The distribution for the null model (χ^2^ distribution with 41 degrees of freedom) is also shown. The distribution for the HL comparison (Fig. 1a) is shifted to very much higher values than expected under the null hypothesis. Most real vales of 2Δ are 200 or higher, for which the *p* value is essentially zero. A *p* value of 5% corresponds to 2Δ = 56.94 (shown as a vertical line). The measured 2Δ exceeds this for every single species, which confirms that there is strong selection on codon usage in high expression genes in bacteria.

The same distribution is shown for the CV comparison in Fig. 1b. The distribution is again shifted noticeably from the null, although not so much as for the HL comparison. In the CV case, 80% of species have a 2Δ value that is significant at the 5% level. Thus, a difference in codon frequencies between the conserved and variable sites on the high expression genes is detectable by this method in the majority of species.

### Variation of the Strength of Codon Bias Among Species

**Figure 2:**
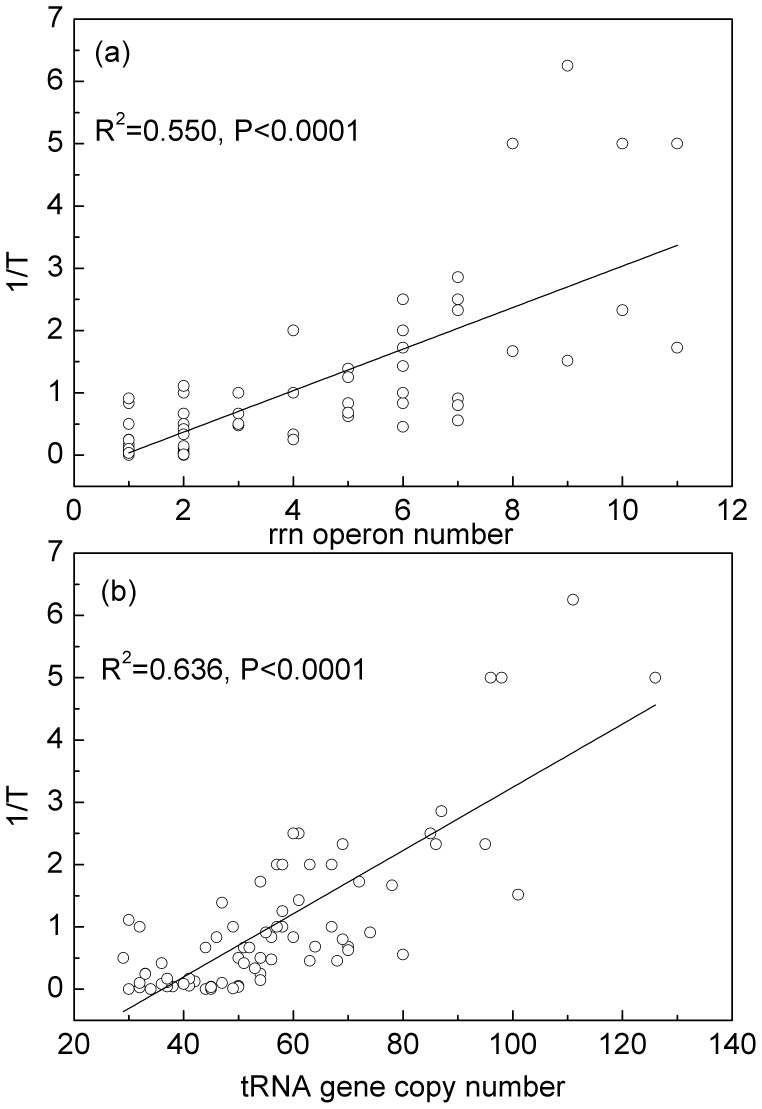
Bacteria growth rate. Growth rate, shown as the inverse of the minimum doubling time, 1/T (hours^−1^), is strongly correlated with the number of copies of (a) ribosomal RNA operons, and (b) tRNA genes.

Ikemura found an organism-specific positive correlation of the usage of cognate codons and respective isoaccepting tRNAs in two organisms [16], [17]. Growth rate in bacteria is positively correlated with RNA-to-protein ratio [18], [19], the total number of copies of ribosomal RNA operons, and the total number of copies of tRNA genes [1], [2]. The latter two are shown in Fig. 2 for the species in our data set. Growth rates are taken from the survey of Rocha [9], and are the observed maximal doubling rate of the species concerned. All these are evidences for speed selection. In this paper, we relate codon bias to total tRNA gene copy number, since there is a wider range of variation among species for tRNAs than for rRNA operons, and because it is easier to quantify and compare between species than growth rate (which depends on experimental conditions). Our conjecture is that if codon bias is strongly correlated with tRNA copy number in a large and diversified species, it is more likely an effect of speed/efficiency since tRNA copy number is a crucial factor for fast growing. Not too much research has been focused on the comparison of both effects from speed and accuracy in a large amount of organisms systematically, might because of a lack of a powerful tool like *δ* that is in log-likelihood units, which means that it is comparable *not only within a genome but also across organisms*. This is a novel contribution to causes of codon bias in bacteria and adds a new content for speed hypothesis as well.

**Figure 3:**
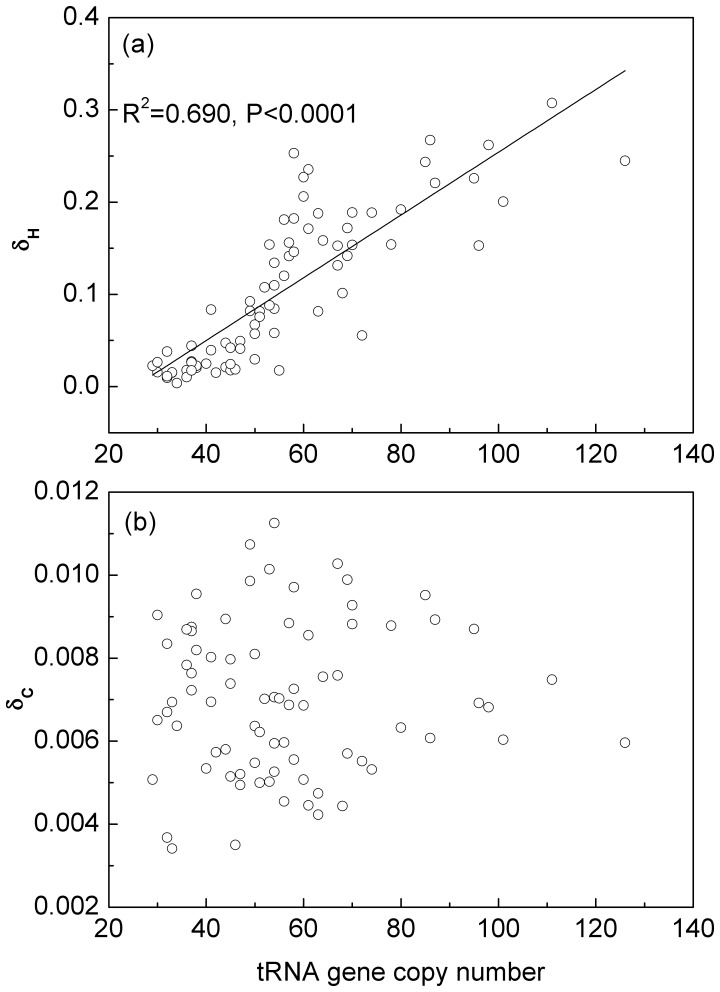
Codon bias in high expression genes and conserved sites. (a) There is a strong positive correlation between strength of codon bias in high expression genes, δ_H_, and tRNA gene copy number. (b) There is no correlation between the strength of bias in conserved sites, δ_C_, and tRNA gene copy number. The fact that δ_C_ is much smaller than δ_H_ suggests that selection for translational speed is more important than accuracy in these bacteria.

Figure 3a shows that the strength of codon bias in high expression genes relative to the rest of the genome, as measured by *δ_H_* (defined in the methods section), is strongly correlated with the number of tRNA gene copies in the genome (r = 0.831, p<0.0001). However, Fig. 3b shows that the values of *δ_C_* are very much smaller than those of *δ_H_*, and there is no correlation between *δ_C_* and tRNA gene copy number. The scale of *δ_H_* can be interpreted in terms of likelihood ratios. For a typical species with *δ_H_* = 0.3, the likelihood improves by a factor of exp(0.3) = 1.35 per codon, when the difference between high and low expression genes is included in the statistical model. For a sequence of 100 codons, this is a factor of exp(0.3×100) = 10^13^, *i.e.* a large effect. However, for *δ* = 0.02, which corresponds to the smallest values observed in *δ_H_* and the largest values observed in *δ_C_*, the likelihood improves only by a factor of exp(0.02) = 1.02 per codon, or 7.4 for a sequence of 100 codons.

Our interpretation is that species that are under selection for fast growth need all available methods for optimizing translation. Thus codon usage is strongly selected for translational efficiency in species where duplicate rRNAs and tRNAs are selected. The difference in codon usage between conserved and variable sites cannot be explained by translational efficiency and is thought to be a signature of selection for translational accuracy. Our results show that selection for accuracy does occur, because the CV comparison gives a significant deviation from the null hypothesis for many species (Fig. 1b). However, the calculation of *δ_C_* (Fig. 3b) shows that this effect is weak in all species and does not correlate with tRNA copy number. Therefore_,_ our conclusion is that the major effect causing biased codon usage in high expression genes in bacteria is selection for translational speed, and that selection for accuracy plays a small supplementary role.

### Comparison of CAI and δ

CAI [3] is frequently used as a measure of codon bias, and has proven useful as a way of distinguishing which genes in an organism are under strongest translational selection. CAI is often correlated with both mRNA abundance and protein abundance [20], [21], [22], [23], [24], [15]. In the calculation of CAI (see Eqn. 7), each codon has a weighting factor that depends only on the codon frequencies in the high expression data set, and is highest for the codon that is most frequent in the high expression set. In contrast, in the quantity *δ* that we proposed in Eqn 6, the weighting factor depends on both the frequency in the high expression set and in the rest of the genome, and is highest for the codon that increases in frequency the most in the high expression set relative to the rest of the genome. A codon that is frequent in high expression genes might be frequent throughout the genome due to mutational bias. If a codon increases in frequency in the high expression set, this is an indication of selection in the high expression genes. Thus *δ* distinguishes more carefully between biases caused by mutation and those caused by selection. In bacteria, the genomic G+C content ranges from 13% to 75% [25], [26] which has a significant influence on CAI values. A further advantage of *δ* is that it is associated with a statistical test, which is not true for CAI. The fact that the scale for *δ* is in log-likelihood units means that it is comparable not only within a genome but also across organisms, whereas the CAI scale is different for each organism and is more difficult to compare. Because of these advantages, *δ* might be also preferable to several methods for codon bias [27], [28]. Gingold and Pilpel [29] reviewed popular methods for codon bias, where no methods can be of high quantification on discrimination between translation efficiency of individual codons and complexity of implementation for many species. Now our new method can.

**Figure 4:**
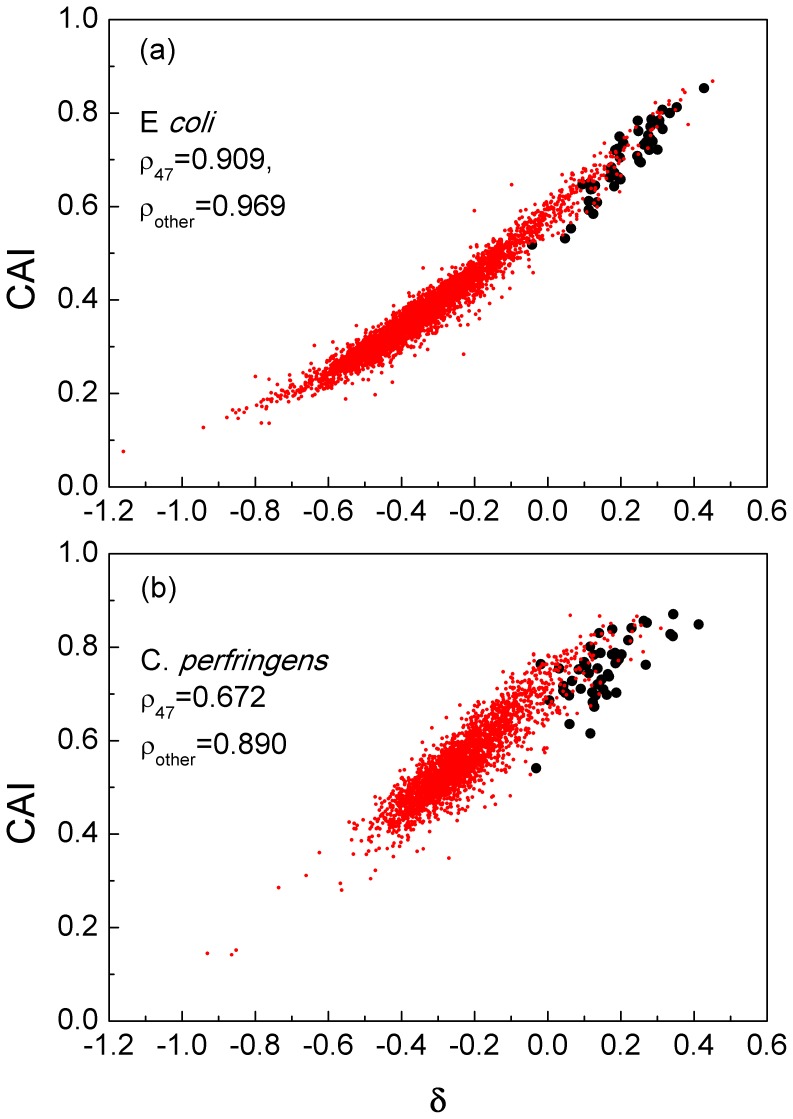
Variation in codon bias among genes in *E. coli* and *C. perfringens*, as measured by CAI and *δ*. Large black points - 47 high-expression reference genes. Small red points - all other genes. ρ is the spearman rank correlation coefficient.

With these points in mind, it is interesting to compare the distributions of CAI and *δ* across genes. Figure 4, shows the example of *E. coli*, chosen because it is a model organism known to have strong codon bias, and *Clostridium perfringens,* chosen because it has a very strong codon bias in the study of Sharp *et al.* [5], a very fast growth rate, and a very low GC content (unlike *E. coli*) and it is not closely related to *E. coli*.

For *E. coli* the two measures are very strongly correlated. The spearman rank correlation coefficient ρ is 0.909 for reference set (large black points); while 0.969, for all other genes. This indicates that genes singled out by CAI would also be singled out by *δ*, and so the two measures are useful for the same purposes. For *C. perfringens*, the correlation is still clear, but less strong: ρ is just 0.672 for reference set (large black points); while 0.890, for all other genes. The separation between the reference set and the rest of the genes is greater with *δ* than with CAI, which suggests that *δ* is slightly improved as a measure. The scale of *δ* is also convenient because positive and negative values of *δ* indicate genes that are or are not adapted to rapid expression in the same way as ribosomal proteins. This same distinction can be used for any organism. For CAI, there is no obvious cutoff between high and low levels.

Table S1 gives the full table of codon frequencies and weighting factors for these two organisms. The codon that is most preferred by selection is usually the one that is most frequent in the high expression genes, but this is not always the case. In *E. coli*, the only exception is the Lys AAA codon, which is most frequent, but under slightly negative selection in high expression genes (*i.e.* 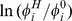 is negative). In *C. perfringens*, there are three exceptions: AUA, GAU, and GGA are all the most frequent codons for their amino acid but are under negative selection. A CAI weighting of 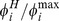 = 1 can mean different things for different codons. For example, in *C. perfringens*, UAC and AAC are under strong positive selection according to 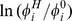, AAA is almost neutral and GAU is under negative selection, but all of these have a weighting of 1. Moderate values of the CAI weighting can also mean different things. For example, in *E. coli*, the codons GUA, GCA, GAU, and GGC all have 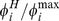 between 0.5 and 0.6, but the first two are under positive selection and the second two are under negative selection.

## Conclusions

This study was intended as a means to compare selection for translational speed and translational accuracy in the same sequences. For this reason we needed a test that works in the same way for the two quantities. The likelihood ratio method that we proposed is able to test the significance of the difference in frequencies between any two sets of codons, and also to measure the strength of the deviation in frequencies on a per-codon basis. It is suitable for comparison of different genomes with different GC contents and mutational biases, and for analysis of biases arising from different causes. In the final part of the paper, we considered the merits of CAI and *δ* as a means of identifying genes under translational selection. Although there is strong correlation between the genic values of CAI and *δ*, we suggest that using *δ* is preferable, especially in organisms of low GC content (like *C. perfringens*) where the mutation bias and selection pressure often favor different codons. Further advantages of *δ* are that it has a log-likelihood scale that is comparable across different organisms, and that it is associated with a simple statistical test for the significance of codon bias.

The difference between frequencies in high and low expression genes is a well-known effect that is highly significant in our statistical method and which is strong enough to be seen with a simple χ^2^ test on one codon family (as in Table 1). The difference between the conserved and variable sites is less pronounced, and has just been tested in a few species previously. It is difficult to see in a test involving one codon family. But when the codon families are combined in the likelihood ratio test that we used here, it is clear that this effect is present in the majority of the bacterial species that we studied. So even a weak signal can be detected by *δ,* which is the reason why we believe our measure is more powerful compared with other methods. The difference between conserved and variable sites is nevertheless small, as shown in Fig. 3. We interpret this as a weak selection for accuracy acting on top of a strong selection for speed and efficiency in the high expression genes.

It is clear that selection for speed will be strongest in those organisms that are adapted to fast growth because time saved in protein production directly effects the cell growth rate and hence its division time. The observed correlations between codon bias (*δ_H_*), growth rate and the number of copies of tRNAs and rRNAs can all be simply explained as results of the same selection pressure for translational speed and efficiency. However, it could also be argued that if selection for accuracy were acting as a result of toxicity of misfolded mistranslated proteins, then the effect would be strongest for highly expressed genes because these genes would produce more misfolded proteins [11]. This might then be an alternative explanation of why selection on codon bias is strongest in highly expressed genes. Could it then be possible that the strong difference in codon frequencies between high and low expression genes that we have been interpreting as due to selection for speed is actually due to selection for accuracy after all? We think not. First, while it is clear that tRNA duplications can speed up translation, it is not clear that they increase accuracy. An overall duplication of all tRNAs would increase correct and incorrect pairing rates proportionally, and should not influence the accuracy. A duplication of a single tRNA might increase the accuracy of translation of the cognate codons but also increase the mispairing rate with near-cognate ones. Thus it is not clear whether a single duplication would be beneficial in terms of accuracy. Furthermore, selection for speed can explain duplication of rRNA operons because this allows rapid production of larger number of ribosomes, whereas increasing the number of ribosomes would not affect the accuracy. Finally, the small effect of accuracy selection that we see between conserved and variable sites is not correlated with tRNA copy number or growth rate (Fig. 3b), which supports the view that accuracy selection is not responsible for the large effect and clear correlation in Fig. 3a. We therefore keep to our conclusion that the main effect seen in the high expression genes is from selection for speed.

In summary, we have introduced a method to detect and quantify codon biases among genomes with different GC content and different mutational biases. Our method is associated with a statistical test that is able to detect a weak signal. The parameter *δ* is comparable not only within a genome but also across organisms. By applying this method to a large range of organisms, we have shown that translational selection is widespread across the bacterial domain and we have helped shed light on the relative importance of the two major factors contributing to codon bias.

## Supporting Information

Table S1 Codon frequencies and weighting factors for *E*. *coli* and *C. perfringens.*
(PDF)
