# Supplementary material for: Contributions of Speed and Accuracy to Translational Selection in Bacteria
Source: PLoS One. 2012 Dec 14;7(12):e51652. doi: 10.1371/journal.pone.0051652 (PMC3522724; doi:10.1371/journal.pone.0051652)
Supplement: Table S1 — Codon frequencies and weighting factors for E. coli and C. perfringens. (PDF) [file pone.0051652.s001.pdf]

E. coli

Codon	Amino acid	ϕ_i^H	ϕ_i^0	$\phi_i^H / \phi_i^{\max}$	$\ln(\phi_i^H / \phi_i^0)$	Anticodon	tRNA copies
UUU	Phe	0.228	0.574	0.295	-0.922	AAA	0
UUC	Phe	0.772	0.426	1.000	0.594	GAA	2
UUA	Leu	0.024	0.131	0.029	-1.688	UAA	1
UUG	Leu	0.040	0.128	0.048	-1.158	CAA	1
CUU	Leu	0.048	0.104	0.057	-0.764	AAG	0
CUC	Leu	0.038	0.104	0.045	-1.004	GAG	1
CUA	Leu	0.002	0.037	0.002	-2.901	UAG	1
CUG	Leu	0.847	0.497	1.000	0.534	CAG	4
AUU	Ile	0.249	0.508	0.332	-0.713	AAU	0
AUC	Ile	0.749	0.420	1.000	0.578	GAU	3
AUA	Ile	0.002	0.072	0.003	-3.446	UAU	0
GUU	Val	0.520	0.259	1.000	0.697	AAC	0
GUC	Val	0.078	0.216	0.150	-1.020	GAC	2
GUA	Val	0.278	0.154	0.535	0.593	UAC	5
GUG	Val	0.124	0.371	0.238	-1.098	CAC	0
UCU	Ser	0.408	0.145	1.000	1.031	AGA	0
UCC	Ser	0.257	0.149	0.631	0.546	GGA	2
UCA	Ser	0.031	0.123	0.077	-1.370	UGA	1
UCG	Ser	0.013	0.154	0.031	-2.508	CGA	1
CCU	Pro	0.142	0.158	0.197	-0.111	AGG	0
CCC	Pro	0.011	0.124	0.016	-2.376	GGG	1
CCA	Pro	0.126	0.191	0.176	-0.413	UGG	1
CCG	Pro	0.720	0.527	1.000	0.313	CGG	1
ACU	Thr	0.468	0.166	1.000	1.037	AGU	0
ACC	Thr	0.442	0.435	0.945	0.016	GGU	2
ACA	Thr	0.049	0.131	0.105	-0.981	UGU	1
ACG	Thr	0.041	0.268	0.088	-1.870	CGU	2
GCU	Ala	0.462	0.161	1.000	1.053	AGC	0
GCC	Ala	0.082	0.270	0.177	-1.196	GGC	2
GCA	Ala	0.269	0.214	0.582	0.229	UGC	3
GCG	Ala	0.188	0.356	0.408	-0.637	CGC	0
UAU	Tyr	0.238	0.569	0.312	-0.873	AUA	0
UAC	Tyr	0.762	0.431	1.000	0.571	GUA	3
CAU	His	0.299	0.572	0.426	-0.649	AUG	0
CAC	His	0.701	0.428	1.000	0.493	GUG	1
CAA	Gln	0.197	0.347	0.245	-0.568	UUG	2
CAG	Gln	0.803	0.653	1.000	0.207	CUG	2
AAU	Asn	0.123	0.451	0.140	-1.303	AUU	0
AAC	Asn	0.877	0.549	1.000	0.469	GUU	4
AAA	Lys	0.715	0.766	1.000	-0.069	UUU	6
AAG	Lys	0.285	0.234	0.398	0.197	CUU	0
GAU	Asp	0.359	0.627	0.560	-0.559	AUC	0
GAC	Asp	0.641	0.373	1.000	0.543	GUC	3
GAA	Glu	0.763	0.690	1.000	0.101	UUC	4

GAG	Glu	0.237	0.310	0.310	-0.270	CUC	0
UGU	Cys	0.316	0.445	0.462	-0.342	ACA	0
UGC	Cys	0.684	0.555	1.000	0.209	GCA	1
CGU	Arg	0.683	0.380	1.000	0.586	ACG	4
CGC	Arg	0.302	0.400	0.442	-0.280	GCG	0
CGA	Arg	0.003	0.064	0.005	-2.951	UCG	0
CGG	Arg	0.005	0.098	0.007	-2.970	CCG	1
AGU	Ser	0.047	0.151	0.115	-1.169	ACU	0
AGC	Ser	0.245	0.277	0.600	-0.125	GCU	1
AGA	Arg	0.007	0.037	0.010	-1.711	UCU	1
AGG	Arg	0.000	0.021	0.002	-3.446	CCU	1
GGU	Gly	0.621	0.338	1.000	0.610	ACC	0
GGC	Gly	0.359	0.404	0.578	-0.117	GCC	4
GGA	Gly	0.006	0.108	0.010	-2.813	UCC	1
GGG	Gly	0.013	0.151	0.021	-2.454	CCC	1

C. perfringers

Codon	Amino acid	ϕ_i^H	ϕ_i^0	$\phi_i^H / \phi_i^{\max}$	$\ln(\phi_i^H / \phi_i^0)$	Anticodon	tRNA copies
UUU	Phe	0.231	0.805	0.301	-1.246	AAA	0
UUC	Phe	0.769	0.195	1	1.37	GAA	4
UUA	Leu	0.762	0.661	1	0.143	UAA	4
UUG	Leu	0.005	0.04	0.007	-1.989	CAA	1
CUU	Leu	0.176	0.205	0.23	-0.154	AAG	0
CUC	Leu	0	0.005	0.002	-3.939	GAG	1
CUA	Leu	0.057	0.085	0.074	-0.401	UAG	3
CUG	Leu	0	0.005	0.002	-3.939	CAG	0
AUU	Ile	0.101	0.331	0.196	-1.182	AAU	0
AUC	Ile	0.381	0.045	0.737	2.129	GAU	4
AUA	Ile	0.517	0.624	1	-0.188	UAU	0
GUU	Val	0.634	0.522	1	0.193	AAC	0
GUC	Val	0.004	0.021	0.007	-1.61	GAC	0
GUA	Val	0.341	0.401	0.538	-0.16	UAC	4
GUG	Val	0.021	0.056	0.033	-0.999	CAC	0
UCU	Ser	0.149	0.257	0.265	-0.547	AGA	0
UCC	Ser	0	0.037	0.002	-3.939	GGA	1
UCA	Ser	0.563	0.324	1	0.552	UGA	2
UCG	Ser	0.007	0.006	0.012	0.119	CGA	0
CCU	Pro	0.254	0.432	0.343	-0.53	AGG	0
CCC	Pro	0	0.02	0.002	-3.939	GGG	0
CCA	Pro	0.742	0.534	1	0.329	UGG	2
CCG	Pro	0.004	0.014	0.005	-1.349	CGG	0
ACU	Thr	0.572	0.505	1	0.125	AGU	0
ACC	Thr	0.002	0.061	0.004	-3.223	GGU	1
ACA	Thr	0.426	0.418	0.745	0.017	UGU	4
ACG	Thr	0	0.016	0.002	-3.939	CGU	0
GCU	Ala	0.654	0.538	1	0.195	AGC	0

GCC	Ala	0.007	0.076	0.011	-2.355	GGC	0
GCA	Ala	0.32	0.365	0.489	-0.133	UGC	6
GCG	Ala	0.019	0.021	0.029	-0.105	CGC	0
UAU	Tyr	0.311	0.836	0.451	-0.989	AUA	0
UAC	Tyr	0.689	0.164	1	1.433	GUA	3
CAU	His	0.429	0.808	0.75	-0.634	AUG	0
CAC	His	0.571	0.192	1	1.091	GUG	2
CAA	Gln	0.966	0.862	1	0.114	UUG	2
CAG	Gln	0.034	0.138	0.035	-1.398	CUG	0
AAU	Asn	0.319	0.824	0.468	-0.949	AUU	0
AAC	Asn	0.681	0.176	1	1.353	GUU	4
AAA	Lys	0.727	0.696	1	0.045	UUU	7
AAG	Lys	0.273	0.304	0.375	-0.11	CUU	2
GAU	Asp	0.614	0.871	1	-0.351	AUC	0
GAC	Asp	0.386	0.129	0.63	1.101	GUC	3
GAA	Glu	0.815	0.77	1	0.057	UUC	3
GAG	Glu	0.185	0.23	0.226	-0.218	CUC	0
UGU	Cys	0.721	0.794	1	-0.096	ACA	0
UGC	Cys	0.279	0.206	0.388	0.303	GCA	2
CGU	Arg	0.026	0.034	0.027	-0.266	ACG	1
CGC	Arg	0	0.003	0.002	-3.939	GCG	0
CGA	Arg	0.002	0.006	0.002	-1.15	UCG	1
CGG	Arg	0	0.001	0.002	-3.939	CCG	0
AGU	Ser	0.113	0.29	0.2	-0.945	ACU	0
AGC	Ser	0.169	0.087	0.3	0.669	GCU	3
AGA	Arg	0.971	0.863	1	0.118	UCU	3
AGG	Arg	0.002	0.094	0.002	-3.939	CCU	1
GGU	Gly	0.397	0.281	0.72	0.344	ACC	0
GGC	Gly	0.047	0.052	0.086	-0.088	GCC	4
GGA	Gly	0.551	0.586	1	-0.061	UCC	7
GGG	Gly	0.005	0.081	0.008	-2.877	CCC	0
